# Subset selective search on the basis of color and preview

**DOI:** 10.3758/s13414-016-1211-7

**Published:** 2016-09-23

**Authors:** Mieke Donk

**Affiliations:** Department of Cognitive Psychology, Vrije Universiteit, Van der Boechorststraat 1, 1081 BT Amsterdam, The Netherlands

**Keywords:** Search, Visual search, Attention: Selective

## Abstract

In the preview paradigm observers are presented with one set of elements (the irrelevant set) followed by the addition of a second set among which the target is presented (the relevant set). Search efficiency in such a preview condition has been demonstrated to be higher than that in a full-baseline condition in which both sets are simultaneously presented, suggesting that a preview of the irrelevant set reduces its influence on the search process. However, numbers of irrelevant and relevant elements are typically not independently manipulated. Moreover, subset selective search also occurs when both sets are presented simultaneously but differ in color. The aim of the present study was to investigate how numbers of irrelevant and relevant elements contribute to preview search in the absence and presence of a color difference between subsets. In two experiments it was demonstrated that a preview reduced the influence of the number of irrelevant elements in the absence but not in the presence of a color difference between subsets. In the presence of a color difference, a preview lowered the effect of the number of relevant elements but only when the target was defined by a unique feature within the relevant set (Experiment 1); when the target was defined by a conjunction of features (Experiment 2), search efficiency as a function of the number of relevant elements was not modulated by a preview. Together the results are in line with the idea that subset selective search is based on different simultaneously operating mechanisms.

New objects can be prioritized over old objects (Donk & Theeuwes, 2001; Jiang, Chun, & Marks, 2002b; Watson & Humphreys, 1997). Major evidence stems from studies using the preview paradigm (Watson & Humphreys, 1997). In this paradigm, observers are usually presented with a modified color-form conjunction search task in which one set of elements (e.g., green Hs) is displayed prior to a second set of elements (e.g., blue As). Observers have the task to search for a pre-specified target (e.g., a blue H) which can only be presented among the second set of elements. The results typically show that search efficiency is much higher in this preview condition than in a full-baseline condition in which all elements are simultaneously presented, a difference denoted as the preview benefit. In fact, search in the preview condition has often been found to be as efficient as that in a half-baseline condition in which only the second set of elements is presented. These results show that observers are able to search for the target among the second set of elements as if the first set of elements is not presented.

Since Watson and Humphreys (1997), numerous studies have been performed to investigate the mechanism underlying prioritized selection in preview search. However, the results of these studies still have not resulted in one unambiguous account of the preview benefit. Watson and Humphreys (1997) originally argued that new elements are prioritized because observers apply top-down inhibition to the old elements (see also Humphreys, Stalmann, & Olivers, 2004; Kunar, Humphreys, Smith, & Watson, 2003; Watson & Humphreys, 2000), a process they referred to as visual marking. This view is based on the idea that during the preview observers are actively involved in the application of inhibition at the old elements’ locations. As a consequence, upon the appearance of the new elements, the old elements no longer compete for attentional resources.

Subsequent studies (e.g., Braithwaite & Humphreys, 2003; Braithwaite, Humphreys, & Hodsoll, 2003; Braithwaite, Humphreys, & Hulleman, 2005; Kunar, Humphreys, Smith, & Hulleman, 2003; Kunar, Humphreys, Smith, & Watson, 2003; Olivers & Humphreys, 2002, 2003; Olivers, Humphreys, & Braithwaite, 2006; Watson & Humphreys, 1998) proved the location-based visual marking account to be incomplete. For instance, Watson and Humphreys (1998) showed that a preview benefit could also be obtained with moving stimuli. To account for this result, it was suggested that apart from location-based inhibition, observers may apply feature-based inhibition to enhance selectivity for the new relevant set of elements. That is, inhibition may not only be applied to specific locations, but to whole feature maps in order to prioritize selection of the relevant elements. Support for this feature-inhibition account with static stimuli came, among others, from a study of Braithwaite and Humphreys (2003; see also Braithwaite & Humphreys, 2007; Braithwaite et al., 2003; Braithwaite, Humphreys, & Hodsoll, 2004; Braithwaite, Humphreys, Hulleman, & Watson, 2007; Olivers & Humphreys, 2003; Olivers et al., 2006; Osugi & Kawahara, 2013). They examined the effects of color mixing across old and new elements on preview search. Color mixing was found to have a profound effect on search efficiency. Performance was worse when the target shared its color with the majority of the previewed irrelevant elements relative to when it shared its color with the minority of the previewed irrelevant elements. These findings suggest that the color of the previewed elements was inhibited to prioritize the selection of the new relevant elements.

A completely different account was put forward by Donk and Theeuwes (2001). They claimed that in preview search it is sufficient for the prioritized selection of new elements to let these new elements be accompanied by abrupt onsets. According to this view, new elements are automatically prioritized over old ones because attention is captured by the luminance onsets that accompany their appearance (e.g., Jonides & Yantis, 1988; Theeuwes, 1991, 1992, 1994; Yantis, 1993; Yantis & Jonides, 1984). Thus, according to this onset capture view, new elements are prioritized in a bottom-up fashion without the involvement of any voluntary processes. In Donk and Theeuwes, observers had to perform a preview search task in which old and new elements were presented either with or without onsets. The results showed that new elements were prioritized only when these were presented with abrupt onsets. When new elements were presented isoluminant with the background, i.e. without onsets, there was no prioritization of new elements. Apparently, onsets were crucial for new elements to receive priority over old elements (see also Agter & Donk, 2005; Belopolsky, Theeuwes, & Kramer, 2005; Donk, 2006; Donk & Theeuwes, 2001, 2003; Donk & Verburg, 2004; Theeuwes, Kramer, & Atchley, 1998; but see Braithwaite, Hulleman, Watson, & Humphreys, 2006; Braithwaite, Humphreys, Watson, & Hulleman, 2005; Braithwaite, Watson, Andrews, & Humphreys, 2010).

A third account was postulated by Jiang et al. (2002b). According to this view the prioritization of new elements is the result of temporal grouping. The old and new elements form two distinct groups on the basis of their temporal asynchrony. After the grouping process, observers can selectively attend to the subset of elements that is relevant to the search task. In the study of Jiang et al., observers were presented with a preview search task in which the target could be embedded either in the set of old elements or in the set of new elements. When observers were informed about which subset contained the target, they could selectively prioritize the target-relevant subset, irrespective of whether it was the subset of old elements or the subset of new elements. The account of Jiang et al. does not speak directly to whether prioritized selection is accomplished via inhibition or some other process, and whether onsets are important to segregate the temporally distinct elements. However, like a visual marking account, this account also assumes that subset selection is top-down in nature. This view differs from the other views with respect to the flexibility to attend to either the old or the new elements. According to the visual marking view and the onset capture view, only the new elements can be prioritized for selection over the old ones, but not vice versa. In the temporal grouping account of Jiang et al., it is assumed that either subset can be prioritized if necessary.

The three accounts put forward to explain the preview benefit differ substantially (see for reviews: Donk, 2006; Humphreys, Hodsoll, Olivers, & Yoon, 2006; Olivers et al., 2006; Watson, Humphreys, & Olivers, 2003). However, they are not mutually exclusive. Even though it is possible that observers always use one prioritization mechanism at a time, it is also possible that observers use multiple mechanisms simultaneously in prioritizing the selection of new over old elements. For example, it is feasible that through the application of top-down inhibition observers may enhance the power of luminance onsets to capture attention (Donk, 2006; Olivers et al., 2006; Watson & Humphreys, 1997). Moreover, in addition to the mechanisms specifically proposed to account for the preview benefit, observers may also use prioritization mechanisms that are not unique to preview search. Also without a preview, observers can selectively attend to one subset of elements and ignore the other one (e.g., Egeth, Virzi, & Garbart, 1984; Friedmanhill & Wolfe, 1995; Kaptein, Theeuwes, & Vanderheijden, 1995; Koshino, 2001). For instance, Kaptein et al. (1995) demonstrated that when observers search for the presence of a target defined by a conjunction of color and orientation, they are capable of restricting search to only those elements that share the target color. More specifically, Kaptein et al. (1995) showed that in search for a red vertical line among a variable number of red tilted lines and green vertical lines, reaction time (RT) was independent of the number of green lines. Thus, the presence of a color difference between relevant and irrelevant sets of elements may, similar to a preview, lead to prioritized selection of the relevant set only, even to the extent that the number of irrelevant elements does no longer affect RT.

Given the finding that people can be highly selective on the basis of color in a standard conjunction search task, the question arises why so many studies have shown that an additional preview in such a task leads to even better performance (e.g., Braithwaite & Humphreys, 2003; Humphreys et al., 2004; Humphreys, Watson, & Jolicoeur, 2002; Irwin & Humphreys, 2013; Kunar, Humphreys, & Smith, 2003a; Olivers & Humphreys, 2003; von Muhlenen, Watson, & Gunnell, 2013; Watson, Compton, & Bailey, 2011; Watson & Humphreys, 1997, 2002; Watson & Inglis, 2007; Watson & Kunar, 2010; Zupan, Watson, & Blagrove, 2015). That is, if irrelevant elements can already be excluded from a search when they carry an irrelevant color, how then can an additional preview lead to any further performance improvements?

There are basically two possibilities. First, subset selection on the basis of color alone might not always be perfect. Consequently, the addition of a preview may result in a better ability to restrict search to the relevant elements which in turn leads to higher search efficiency in preview compared to full-baseline conditions. This might be particularly the case when the colors discriminating the relevant from the irrelevant elements are similar (Nagy & Sanchez, 1990; Wolfe, Cave, & Franzel, 1989), which is often the case in preview experiments (e.g., Kunar, Humphreys, & Smith, 2003b; Olivers, Watson, & Humphreys, 1999; Watson, 2001; Watson & Humphreys, 1997, 1998). For instance, if observers are asked to search for a blue vertical line among simultaneously presented green verticals and blue horizontal lines, observers might prioritize the task-relevant color, which is blue in this case (e.g., D'Zmura, 1991; Wolfe & Horowitz, 2004). However, this prioritization might not be perfect. That is, the green items might occasionally be falsely selected with the result that search might not be completely independent from the number of green items. Implementing an additional preview of the green elements might subsequently lead to a better prioritization of the new elements either because the preview allows the green elements to be more effectively inhibited (Braithwaite & Humphreys, 2003; Watson & Humphreys, 1997) or because the relevant elements capture attention through their unique onset (Donk & Theeuwes, 2001). Either way, search will become more efficient in the preview condition relative to the full-baseline condition because the irrelevant set will become more effectively excluded from the search process with a preview of the irrelevant green set than without.

Alternatively, it is possible that the addition of a preview makes search through the relevant elements more efficient. The preview benefit may thus not arise as a result of a better ability to exclude the irrelevant ‘old’ elements from search but merely as the consequence of a more efficient search process through the relevant ‘new’ elements. This would imply that the preview benefit reflects a reduction in the influence of the number of relevant rather than irrelevant elements. There are various studies suggesting that search through a color-defined subset of elements is less efficient than search through that same set presented in isolation (Egeth et al., 1984; Friedmanhill & Wolfe, 1995; Kaptein et al., 1995; Theeuwes, 1994). For instance, in the study of Kaptein et al. (1995) in which observers searched for a red vertical line among red tilted lines and green vertical lines, observers ignored the irrelevant set of green elements as evident by their finding that RT was independent of the number of green elements in the display. However, despite the fact that the target was defined by a unique feature relative to the remaining red elements, the target did not pop out: RT increased substantially as a function of the relevant set size. Apparently, subset-selection on the basis of color alone is not strong enough to allow search to proceed in parallel across the elements in the target color (see e.g., Friedmanhill & Wolfe, 1995). However, when irrelevant and relevant subsets are presented in a spatially separated location, search through a color-defined subset can again become very efficient (Theeuwes, 1996). Indeed, in Theeuwes (1996), observers also searched for a red vertical line among red tilted lines and green vertical lines. The red elements were either spatially scattered among the green ones or presented at adjacent positions such that they were spatially separated from the green elements. The results showed that even though observers were well able to ignore the green elements in both conditions, search efficiency as a function of the number of red elements varied strongly across conditions: search through the red subset proceeded in parallel when red elements were grouped whereas this was not the case when red elements were spatially scattered among the green ones. These results suggest that even though the presence of a color difference between irrelevant and relevant sets of elements is not sufficient to evoke parallel search among the relevant elements, the addition of a spatial separation between sets is (see for similar results with motion-color conjunctions and the addition of stereoscopic disparity: Nakayama & Silverman, 1986). It is conceivable that an additional temporal separation brings about a similar effect. Possibly, the addition of a temporal separation as induced by a preview also allows observers to detect a feature-defined target in parallel across the relevant elements. Accordingly, the typically observed preview benefit in the presence of a color difference between relevant and irrelevant sets of elements may be a reflection of a more efficient search process through the relevant elements rather than of the capability to more effectively ignore the irrelevant elements.

It is interesting to note that there are several preview studies in which irrelevant and relevant set size effects were explicitly dissociated (e.g., Al-Aidroos, Emrich, Ferber, & Pratt, 2012; Donk, Agter, & Pratt, 2009; Donk & Theeuwes, 2001; Emrich, Ruppel, Al-Aidroos, Pratt, & Ferber, 2008; Jiang, Chun, & Marks, 2002a; Jiang et al., 2002b; Peterson, Belopolsky, & Kramer, 2003; Theeuwes et al., 1998). However, in these studies both subsets typically had the same color and the target was not defined by a unique feature within the relevant subset of elements. Moreover, the influence of irrelevant and relevant set sizes was generally not compared between preview and half- and full-baseline conditions. These studies therefore never addressed the question whether a preview leads to any change in the manner in which relevant elements are searched. Conversely, preview studies with differently colored subsets and uniquely defined targets within the relevant subset typically compared preview performance to that in a half- and full-baseline condition, but commonly failed to separate the effects of numbers of irrelevant and relevant elements. As a result, observed changes in search efficiency could never unequivocally be attributed to either a better ability to ignore the irrelevant set of elements or a more efficient search process through the relevant elements.

The present study aimed to systematically disentangle the contributions of irrelevant and relevant set sizes to preview search in the absence and the presence of a color difference between subsets of elements. To this aim two experiments were performed in which observers had the task to search for a target among a relevant set of elements and ignore an irrelevant set of elements. Numbers of irrelevant and relevant elements were independently manipulated and either simultaneously presented or separated by a preview of the irrelevant set of elements. Moreover, irrelevant and relevant elements were either presented in one color or were distinctively colored. The orthogonal manipulation of Preview (absent, present) and Color (absent, present) resulted in four different conditions: the no-color full-baseline condition, the no-color preview condition, the color full-baseline condition, and the color preview condition. To allow comparison to a condition without any selection requirements, there was also a fifth condition, the half-baseline condition, in which only the relevant elements were presented. In Experiment 1, the target was defined by a conjunction of line segments relative to the irrelevant set of elements, but by a unique feature relative to the relevant set of elements, potentially allowing parallel search among the relevant elements. This experiment examined whether the presence of a preview can evoke a shift from a serial to a parallel search process through the relevant elements, similar to that observed by Theeuwes (1996). In Experiment 2, the target was defined by a conjunction of line segments relative to both the irrelevant and the relevant set of elements. This experiment examined whether the presence of a preview can enhance the search rate through the relevant elements under conditions in which target detection requires a serial search process.

If on the one hand the preview benefit is based on a better ability to exclude the irrelevant elements from search, the effect of the number of irrelevant elements is predicted to be smaller in the preview compared to the full-baseline conditions. Moreover, the effect of the number of relevant elements is predicted to be equal across conditions. If on the other hand the preview benefit arises as a result of a more efficient search process through the relevant elements, the effect of the number of irrelevant elements is predicted to be equal across the preview and the full-baseline conditions and the effect of the number of relevant elements should be reduced in preview compared to full-baseline conditions. In addition, search efficiency as a function of the number of relevant elements should be equal across the half-baseline and preview conditions but larger in the full-baseline conditions than in the other conditions.

## Experiment 1

### Methods

#### Participants

Twenty-five participants (six males, age range 18–28 years) who took part in the present experiment in exchange for credits or money were included in the analyses. Three further participants (two males, age range 18–22 years) were excluded because their error percentages exceeded 15.0 % (i.e., 20.0 %, 18.5 %, and 17.9 %). All participants had normal or corrected-to-normal vision and did not know the purpose of the experiment. The experiment was conducted in accordance with the guidelines of the Helsinki Declaration.

#### Apparatus

An HP Compaq 6300 Pro SFF PC controlled the timing of events, the generation of the stimuli, and the recording of the responses. Stimuli were presented on a Samsung SyncMaster 2233RZ monitor (47.5 × 29.5 cm, refresh rate: 120 Hz; resolution: 1,680 × 1,050 pixels). The software package E-Prime (Schneider, Eschman, & Zuccolotto, 2002) was used for the lay-out and timing of the experimental trials. Participants were tested in a dimly-lit room while seated approximately 70 cm in front of the monitor. Their index fingers rested on the [z] and [m] keys, which served as the response keys.

#### Task and stimuli

**Fig. 1:**
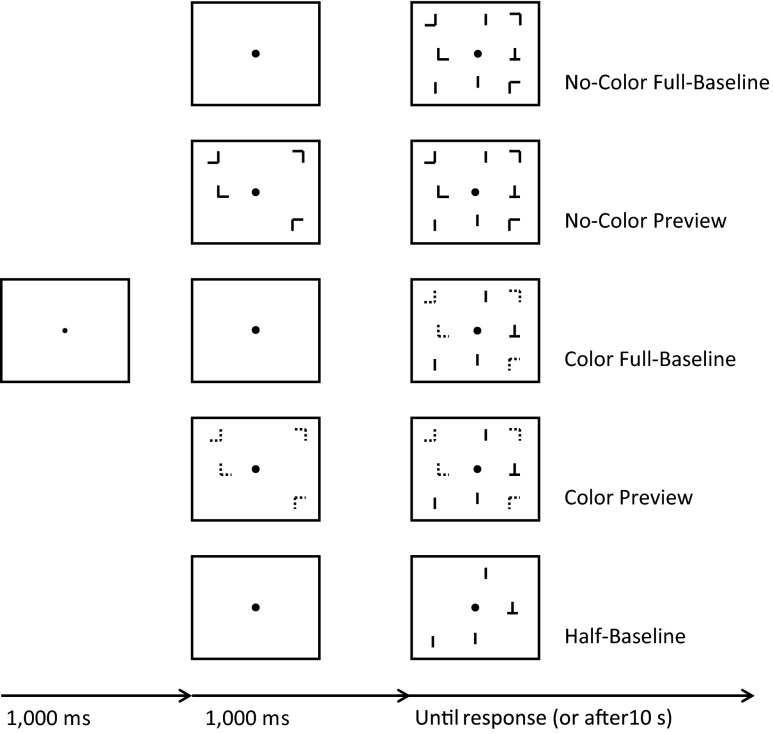
Schematic (unscaled) representation of the displays presented in the five conditions in Experiment 1. For illustrative purposes the numbers of irrelevant and relevant elements in the depicted sample displays are reduced relative to those in the actual experiment. Observers had the task of indicating whether the “T” was upright or upside down. The target is an inverted T in the sample displays. Note that in the actual experiment, the background was black, the central fixation dot was gray, the black elements were green, and the dotted black elements were red (see text)

Participants had to search for a green “T” and indicate whether the T was upright or upside down. Both orientations occurred equally often. Participants had to press the [z] key if the target was an inverted T and the [m] key if the target was an upright T. The target was always embedded within a relevant subset of elements consisting of a variable number of green vertical lines (.95 × .20 cm) rendering the target uniquely distinct within this set of elements. The irrelevant set of elements consisted of a variable number of “Ls” (.95 × .95 cm), which were randomly rotated either 0°, 90°, 180°, or 270°. The stimuli were presented on a black background (0 cd/m^2^). There were five conditions: no-color full-baseline, no-color preview, color full-baseline, color preview, and half-baseline (see Fig. 1). In the *no-color full-baseline* condition, each trial began with a small gray fixation dot (.075 cm × .075 cm, CIE x-, y-chromaticity coordinates of .232, .470, with a luminance of 17.75 cd/m^2^) presented in the middle of the screen. After 1,000 ms the fixation dot increased in size (.15 × .15 cm) to provide a temporal signal which was equal across conditions. Following another 1,000 ms, the irrelevant set of elements consisting of six or 12 green rotated “Ls” (.95 × .95 cm, Commission Internationale de l’Eclairage [CIE] *x*-, *y*-chromaticity coordinates of .260, .661, with a luminance of 16.05 cd/m^2^) and the relevant set consisting of five or 11 green vertical lines (.95 × .20 cm, CIE x-, y-chromaticity coordinates of .260, .661, with a luminance of 16.05 cd/m^2^) and the green target (.95 × .95 cm, Commission Internationale de l’Eclairage [CIE] *x*-, *y*-chromaticity coordinates of .260, .661, with a luminance of 16.05 cd/m^2^) were added to the display. All elements remained on the screen until a response was given or for a maximum of 10 s after target presentation. An erroneous response was followed by a 1,500 Hz tone that was presented for 100 ms. The next trial began 250 ms after the response. The *no-color preview* condition was identical to the no-color full-baseline condition except that the irrelevant set of elements was presented 1,000 ms before the relevant set of elements upon the moment the fixation dot increased in size. The *color full-baseline* condition was identical to the no-color full-baseline condition except that the irrelevant elements were presented in red (Commission Internationale de l’Eclairage [CIE] *x*-, *y*-chromaticity coordinates of .493, 464, with a luminance of 10.73 cd/m^2^) instead of green. The *color preview* condition was identical to the color full-baseline condition except that the irrelevant set of elements was presented 1,000 ms before the relevant set of elements simultaneously with the fixation dot increase. Finally, the *half-baseline* condition was equal to all other conditions, except that only the relevant elements were presented on the screen.

Search displays were generated by positioning each element randomly in one of 35 (7 × 5) invisible matrix cells, which comprised an imaginary rectangle of approximately 28.95 × 21.58 cm, and were presented in synchrony with the screen refresh. The arrangement of the elements was slightly jittered, with the horizontal and vertical centre-to-centre distance between adjacent elements varying between 3.04 and 4.16 cm (mean 3.60 cm).

#### Design and procedure

The five conditions (no-color full-baseline, no-color preview, color full-baseline, color preview, and half-baseline) were blocked, and the specific order in which these conditions were presented was counterbalanced according to a Latin square design across participants. For each participant the specific order of conditions was repeated five times resulting in a total of 25 blocks spread over five cycles with identical block order. A block of trials consisted of 40 trials. The first eight trials of each block served as practice for the remaining of each block in which each combination of Number of Irrelevant Elements, Number of Relevant Elements, and target identity was equally often presented in random order. After each block of trials, participants received feedback about their performance on the screen. By pressing the space bar, they could initiate the next block of trials.

Prior to the experiment, participants were informed about which subset of elements comprised the target, and which subset of elements was irrelevant to the search task. Furthermore, they were instructed to limit their search to the relevant elements only, and to respond as fast as possible without making too many errors.

### Results

Trials with reaction times (RTs) smaller than 300 ms, larger than 6,000 ms, and those with incorrect responses were counted as errors and discarded from further RT analyses. Three participants were replaced because their error percentages were higher than 15.0 % (i.e., 20.0 %, 18.5 %, and 17.9 %). The average error percentage of the remaining participants was 4.2 % (consisting of 0.3 % trials in which a timing error was made, 3.8 % trials in which an incorrect response was given, and 0.1 % trials in which both occurred).

**Fig. 2:**
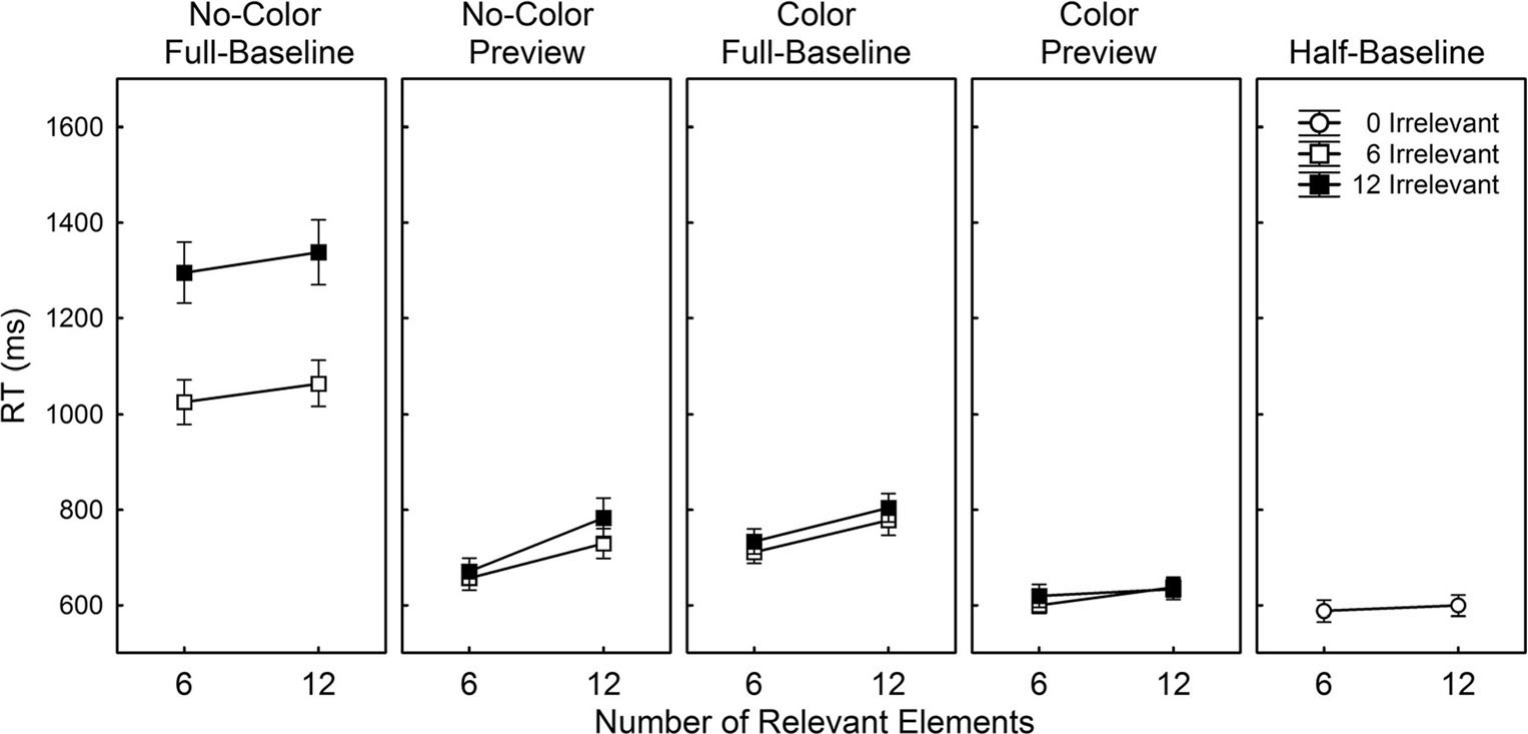
Average reaction time (RT; ms) as a function of the number of irrelevant and relevant elements separately per condition in Experiment 1. Note that the right panel (half-baseline) includes the results obtained in the half-baseline condition in which no irrelevant elements were presented. Error bars reflect the standard error of the mean

Figure 2 shows the averaged RTs over the 25 participants separately per condition. In the following analyses, in those cases in which the assumption of Sphericity was violated (*p* < .05), degrees of freedom were corrected using Greenhouse-Geisser estimates of Sphericity.

To investigate how preview and color contribute to subset-selective search, an ANOVA was conducted on the individual averaged correct RTs obtained in the no-color full-baseline, no-color preview, color full-baseline, and color preview conditions with Color (absent, present), Preview (absent, present), Number of Irrelevant Elements (6, 12), and Number of Relevant Elements (6, 12) as repeated-measures factors. Overall, RT decreased in the presence of a color difference, *F*(1, 24) = 132.91, *MSE* = 49,157, *p* < .001, and in the presence of a preview, *F*(1, 24) = 238.79, *MSE* = 38,275, *p* < .001. Moreover, RT increased as a function of the number of irrelevant elements, *F*(1, 24) = 91.49, *MSE* = 7,773, *p* < .001, and as a function of the number of relevant elements, *F*(1, 24) = 81.24, *MSE* = 3,982, *p* < .001. There was a significant Color × Preview interaction, *F*(1, 24) = 153.19, *MSE* = 18,459, *p* < .001, indicating that the effect of Preview was larger in the absence than in the presence of a color difference.

The effect of the number of irrelevant elements was modulated by Color, *F*(1, 24) = 85.17, *MSE* = 5,477, *p* < .001, indicating that the presence of a color difference contributed to a better ability to exclude the irrelevant elements from search. The effect of the number of irrelevant elements was also reduced by the presence of a preview, *F*(1, 24) = 65.69, *MSE* = 6,140, *p* < .001. Importantly, there was a significant Color × Preview × Number of Irrelevant Elements interaction, *F*(1, 24) = 54.04, *MSE* = 5,665, *p* < .001, which shows that the effect of Preview on the efficiency of search through the irrelevant elements was smaller with than without a color difference between subsets of elements.

Even though the effect of the number of relevant elements was not modulated by Preview, *F* < 1, it was by Color, *F*(1, 24) = 4.57, *MSE* = 2,033, *p* = .043. This finding shows that the effect of the number of relevant elements tends to be smaller with than without a color difference between subsets. However, critically, there was also a significant Color × Preview × Number of Relevant Elements interaction, *F*(1, 24) = 13.18, *MSE* = 4,277, *p* = .001, which demonstrates that the effect of the number of relevant elements is differently modulated by the presence of a preview in the no-color conditions compared to the color conditions. There were no further effects (all *p*’s > .126).

To investigate how the number of relevant elements affected search in the no-color full-baseline, the no-color preview, the color full-baseline, and the color preview conditions relative to the half-baseline condition, the data obtained in these former conditions were collapsed over the two levels of Number of Irrelevant Elements. Planned comparisons showed that the effect of the number of relevant elements was statistically equivalent across the no-color full-baseline and the half-baseline condition, *F*(1, 24) = 2.51, *MSE* = 1,670, *p* = .126, and across the color preview and the half-baseline condition, *F*(1, 24) = 3.64, *MSE* = 378.84, *p* = .069. However, compared to the half-baseline condition, it was significantly larger in the no-color preview condition, *F*(1, 24) = 33.32, *MSE* = 1,255, *p* < .001, and the color full-baseline condition, *F*(1, 24) = 40.14, *MSE* = 515.93, *p* < .001, indicating that search in these latter conditions was more strongly affected by the number of relevant elements than search in the half-baseline condition.

#### Search slopes

Given the finding that Number of Irrelevant Elements did not interact with Number of Relevant Elements, and in order to directly compare the search rates obtained in the different conditions, average search slopes were calculated as a function of the number of irrelevant elements and as a function of the number of relevant elements separately per condition (see Table 1).

**Table 1:** Averaged search slopes (ms/element) as a function of Number of Irrelevant Elements and Number of Relevant Elements, separately calculated for each of the conditions in Experiment 1

	Number (SD) of Irrelevant Elements	Number (SD) of Relevant Elements
No-Color Full-Baseline	45.18 (22.55)	6.17 (12.93)
No-Color Preview	5.54 (8.50)	15.49 (11.43)
Color Full-Baseline	4.03 (6.00)	11.45 (6.39)
Color Preview	1.32 (8.64)	4.33 (5.43)
Half-Baseline	^-^	1.86 (3.79)

An overall ANOVA on the average slopes as a function of the number of irrelevant elements with the variable Condition (no-color full-baseline, no-color preview, color full-baseline, and color preview) showed a significant effect of Condition, *F*(1.48, 35.40) = 68.72, *MSE* = 321.65, *p* < .001, indicating that the contribution of the number of irrelevant elements varied across conditions. Post-hoc comparisons using the Fisher LSD test revealed that the slopes in the no-color preview, the color full-baseline, and the color preview conditions were statistically equivalent to each other (all *p*’s > .239) but less steep than those obtained in the no-color full-baseline condition (all *p*’s < .001).

An ANOVA on the average slopes as a function of the number of relevant elements with the variable Condition (no-color full-baseline, no-color preview, color full-baseline, color preview, and half-baseline) also showed a significant effect of Condition, *F*(2.13, 51.13) = 10.46, *MSE* = 137.39, *p* < .001. Post-hoc comparisons using the Fisher LSD test revealed that the slopes obtained in the no-color preview condition and color full-baseline condition were statistically equivalent (*p* = .098) but steeper than those in the other conditions (all *p*’s < .032). There were no further slope differences between the no-color full-baseline, the color preview, and the half-baseline condition (all *p*'s > .078).

#### Error rates

Table 2 depicts the averaged error rates (%) per condition. The average error rate across conditions was 4.2 %, and the overall pattern of errors resembled the pattern of RTs. Since there was no indication for any speed-accuracy trade-off, the error rates were not further analyzed.

**Table 2:** Average error rates (%) separately per condition in Experiment 1

	Number of Relevant Elements			
	6		12	
Half-Baseline	3.4		3.2	
	Number of Irrelevant Elements		Number of Irrelevant Elements	
	6	12	6	12
No-Color Full-Baseline	4.5	6.1	4.0	8.7
No-Color Preview	4.1	3.7	4.9	5.6
Color Full-Baseline	3.7	3.5	2.9	3.4
Color Preview	3.4	5.0	2.6	3.8

### Discussion

The aim of Experiment 1 was to investigate how irrelevant and relevant set sizes contribute to preview search in the absence and the presence of a color difference between subsets of elements. Experiment 1 was designed such that the target was defined by a unique feature relative to the relevant set of elements so as to examine whether it would be possible to achieve parallel subset selective search in the presence of a preview (Theeuwes, 1996).

The results obtained in the absence of a color difference show that a preview decreased the influence of the number of irrelevant elements but increased the influence of the number of relevant elements relative to the no-color full-baseline condition. The outcome of a reduced influence of the irrelevant set size is in line with previous findings on preview search (see e.g., Al-Aidroos et al., 2012; Donk & Theeuwes, 2001; Emrich et al., 2008; Hodsoll & Humphreys, 2007; Theeuwes et al., 1998) and substantiates the idea that a preview contributes to a better ability to restrict search to the relevant elements. However, the finding that the presence of a preview increased the effect of the number of relevant elements relative to the no-color full-baseline condition is new, and indicates that observers were less efficient to search through the relevant elements in the no-color preview than in the no-color full-baseline condition.

One explanation for this finding might be related to the fact that it took observers on average much longer to find the target in the no-color full-baseline condition than in the no-color preview condition. As a result they might have been more inclined to prematurely terminate the search process which in turn might have led to shallower search slopes. Yet, if this would have been the case, search slopes as a function of the number of relevant elements should have been more shallow in the presence of 12 than in the presence of six irrelevant elements in the no-color full-baseline condition, for RTs were generally much longer with 12 than with six irrelevant elements. However, the results did not show any interaction between numbers of irrelevant and relevant elements. Alternatively, it might have been the case that observers were able to simultaneously reject larger chunks of elements in the no-color full-baseline condition than in the no-color preview condition. The absence of a preview might have somehow increased the perceived uniformity across subsets which in turn might have allowed observers to effectively discard larger chunks of distractors from the search process in the no-color full-baseline condition than in the no-color preview condition. Indeed, there are various studies suggesting that an increase in the similarity between distractors enhances search efficiency in the sense that observers become more capable of simultaneously rejecting larger groups of distractors with increasing distractor homogeneity (see for a similar account also: Duncan & Humphreys, 1989; Hodsoll & Humphreys, 2007; Poisson & Wilkinson, 1992). Possibly, a preview in the absence of a color difference does not only endow observers with the ability to effectively eliminate the irrelevant set of elements from the search process but also takes away their capability to simultaneously ignore larger groups of distractors. It is important to note that the search slope as a function of the number of relevant elements in the no-color preview condition was not only enhanced relative to the no-color full-baseline condition but also relative to the half-baseline condition. This latter finding confirms the idea that the plain presence of a preview prevents search through the relevant elements to proceed in an optimal way.

The effects of the presence of a color difference between subsets were very similar to those of a preview. The presence of a color difference between subsets substantially reduced the influence of the number of irrelevant elements but simultaneously increased the effect of the number of relevant elements relative to the no-color full-baseline condition. More important in this respect is the finding that the search slope as a function of the number of relevant elements in the color full-baseline condition was also substantially steeper than that in the half-baseline condition. This result bears much resemblance to those earlier reported in studies on color selectivity (Egeth et al., 1984; Friedmanhill & Wolfe, 1995; Kaptein et al., 1995; Theeuwes, 1994) and suggests that color-based subset-selective search does not allow search to proceed in parallel across the elements in the relevant set.

The preview results obtained in the presence of a color difference were quite different from those obtained in the absence of a color difference. Even though the effect of the number of irrelevant elements did not reliably differ between the color preview and the color full-baseline condition, search through the relevant elements proceeded much faster in the former than in latter condition. In fact, the search rate as a function of the number of relevant elements obtained in the color preview condition was statistically equivalent to that in the half-baseline condition. Accordingly, search in the presence of both a preview and a color difference was more efficient than search in the presence of a color difference only. However, this preview benefit was not, as suggested by previous studies (Braithwaite & Humphreys, 2003; Humphreys, Olivers, & Braithwaite, 2006; Humphreys et al., 2004; Humphreys et al., 2002; Irwin & Humphreys, 2013; Kiss & Eimer, 2011; Kunar, Humphreys, & Smith, 2003a, 2003b; Kunar, Humphreys, Smith, & Hulleman, 2003; Kunar, Humphreys, Smith, & Watson, 2003; Olivers & Humphreys, 2003; Olivers et al., 1999; von Muhlenen et al., 2013; Watson, 2001; Watson, Braithwaite, & Humphreys, 2008; Watson et al., 2011; Watson & Humphreys, 1997, 1998, 2002; Watson & Inglis, 2007; Watson & Kunar, 2010; Zupan et al., 2015) due to a reduced influence of the irrelevant elements. It was rather the case that the additional presence of a preview led to a more efficient search process through the relevant elements. These findings are similar to those reported by Theeuwes (1996) who found that the implementation of a spatial separation between two differently colored subsets of elements fundamentally changes the search process through the relevant set from a serial to a parallel search mode. The present results suggest that parallel search may also be achieved by the addition of a temporal separation between differently colored sets.

## Experiment 2

The results of Experiment 1 show that a preview in the presence of a color difference leads to a more efficient search process through the relevant elements. However, at this point it is unclear whether this finding is specific for a target defined by a unique feature within the relevant subset. On the one hand it is possible that this result does not depend on the type of search required to distinguish the target from the distractors within the relevant set of elements. That is, the efficiency of search through the relevant set of elements may generally be enhanced by the simultaneous presence of a preview and a color difference compared to the single presence of a color difference. On the other hand, it is viable that this finding is critically dependent on the specific target-distractor relationship within the relevant set of elements. That is, it may only occur when the target is uniquely defined within the relevant set of elements. Experiment 2 aimed to distinguish between these possibilities by using a target which was defined by a conjunction of line segments within the set of relevant elements.

### Methods

#### Participants

Twenty-five participants (three males, age range 18–27 years) who took part in the present experiment in exchange for credits or money were included in the analyses. Three further participants (two males, age range 23–26 years) were excluded because their error percentages exceeded 15.0 % (i.e., 47.1 %, 22.8 %, and 19.5 %). All participants had normal or corrected-to-normal vision and did not know the purpose of the experiment. The experiment was conducted in accordance with the guidelines of the Helsinki Declaration.

#### Apparatus

The materials were identical to those in Experiment 1.

#### Task and stimuli

The task and stimuli were identical to those in Experiment 1 except that rather than only the irrelevant elements, now both the irrelevant and the relevant elements consisted of a variable number of “Ls” (.95 × .95 cm), which were randomly rotated either 0°, 90°, 180°, or 270°.

#### Design and procedure

The design and procedure were the same as those in Experiment 1.

### Results

Trials with RTs smaller than 300 ms, larger than 6,000 ms, and those with incorrect responses were counted as errors and discarded from further RT analyses. Three participants were replaced because their error percentages were higher than 15.0 % (i.e., 47.1 %, 22.8 %, and 19.5 %). The average error percentage of the remaining participants was 6.0 % (consisting of 0.2 % trials in which a timing error was made, 5.6 % trials in which an incorrect response was given, and 0.2 % trials in which both occurred).

**Fig. 3:**
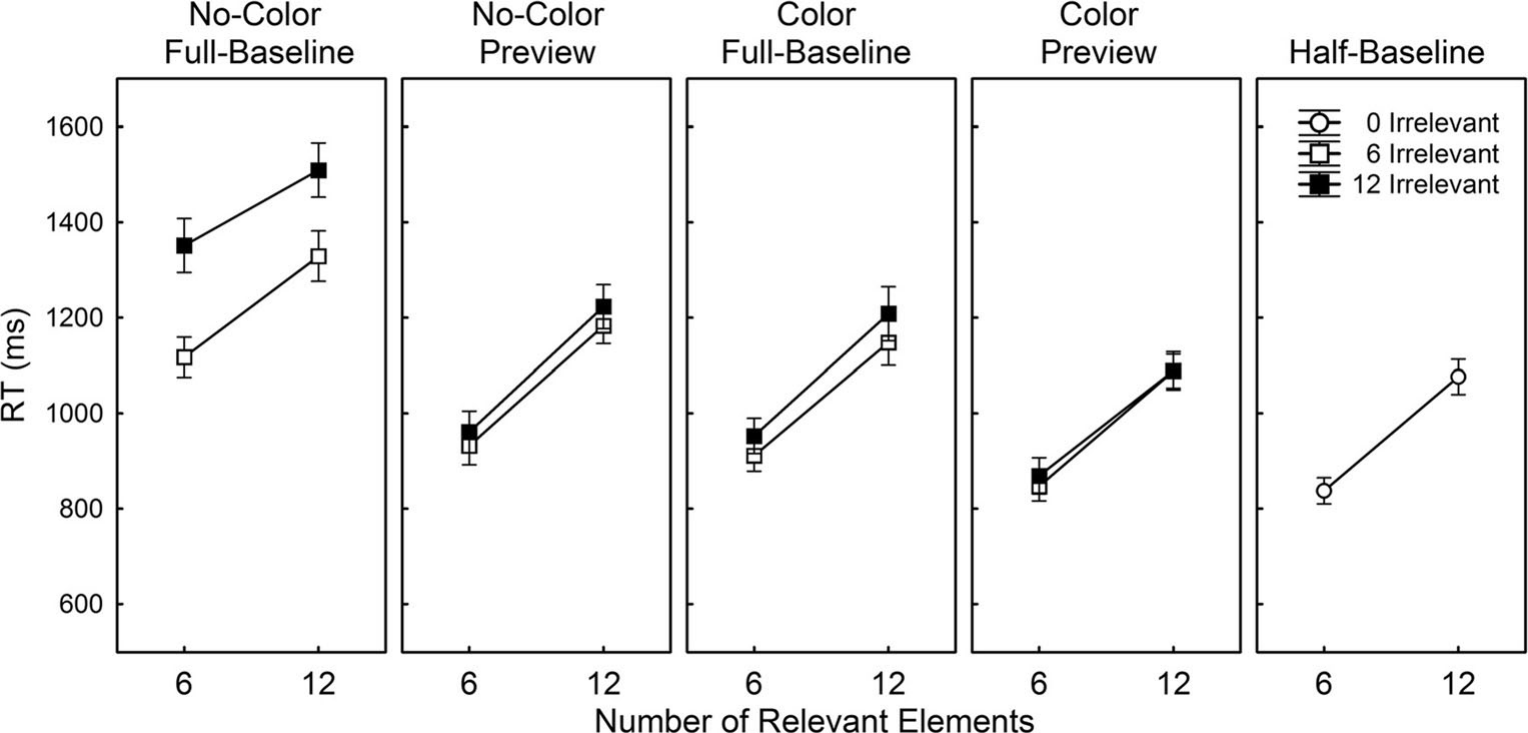
Average reaction time (RT; ms) as a function of the number of irrelevant and relevant elements separately per condition in Experiment 2. Note that the right panel (half-baseline) includes the results obtained in the half-baseline condition in which no irrelevant elements were presented. Error bars reflect the standard error of the mean

Figure 3 shows the averaged RTs over the 25 participants separately per condition.

To examine how a preview and the presence of a color difference between irrelevant and relevant sets of elements affected performance, an ANOVA was conducted on the individual averaged correct RTs obtained in the no-color full-baseline, no-color preview, color full-baseline, and color preview conditions with Color (absent, present), Preview (absent, present), Number of Irrelevant Elements (6, 12), and Number of Relevant Elements (6, 12) as repeated-measures factors. There was a significant effect of Color, *F*(1, 24) = 159.30, *MSE* = 21,822, *p* < .001, Preview, *F*(1, 24) = 51.98, *MSE* = 53,549, *p* < .001, Number of Irrelevant Elements, *F*(1, 24) = 57.29, *MSE* = 10,073, *p* < .001, and Number of Relevant Elements, *F*(1, 24) = 377.47, *MSE* = 13,948, *p* < .001. Generally, RT was lower with a color difference than without, and with a preview than without. Moreover, RT increased as a function of the number of irrelevant elements and as a function of the number of relevant elements. The effect of Preview was larger when irrelevant and relevant elements had the same compared to a different color, *F*(1, 24) = 15.08, *MSE* = 48,138, *p* < .001.

The effect of the number of irrelevant elements was smaller with a color difference than without, *F*(1, 24) = 19.46, *MSE* = 10,378, *p* < .001, and it was smaller with a preview than without, *F*(1, 24) = 26.97, *MSE* = 10,427, *p* < .001. More importantly, there was a significant Color × Preview × Number of Irrelevant Elements interaction, *F*(1, 24) = 14.11, *MSE* = 7,828, *p* = .001, indicating that the effect of Preview on the efficiency of search through the irrelevant elements was larger in the absence than in the presence of a color difference.

The effect of the number of relevant elements was not modulated by the presence of a color difference, *F* < 1, nor by the presence of a preview, *F*(1, 24) = 2.19, *MSE* = 8,925, *p* = .152. However, there was a significant Color × Preview × Number of Relevant Elements interaction, *F*(1, 24) = 5.62, *MSE* = 8,482, *p* = .026, showing that the effect of the number of relevant elements was differently affected by the presence of a preview in the no-color conditions compared to the color conditions. There were no further effects (all *p*’s > .245).

To investigate how the number of relevant elements affected search in the no-color full-baseline, the no-color preview, the color full-baseline, and the color preview conditions relative to the half-baseline condition, the data obtained in these former conditions were collapsed over the two levels of Number of Irrelevant Elements. Planned comparisons showed that the effect of the number of relevant elements was smaller in the no-color full-baseline condition than in the half-baseline condition, *F*(1, 24) = 9.48, *MSE* = 1,966, *p* = .005. Further planned comparisons showed that the effects of the number of relevant elements in the no-color preview condition, the color full-baseline condition, and the color preview conditions were statistically equivalent to that in the half-baseline condition (all *F*’s < 1).

#### Search slopes

Given the finding that Number of Irrelevant Elements did not interact with Number of Relevant Elements, and to compare the search rates obtained in the different conditions, average search slopes were calculated as a function of the number of irrelevant elements and as a function of the number of relevant elements separately per condition (see Table 3).

**Table 3:** Averaged search slopes (ms/element) as a function of Number of Irrelevant Elements and Number of Relevant Elements, separately calculated for each of the conditions in Experiment 2

	Number (SD) of Irrelevant Elements	Number (SD) of Relevant Elements
No-Color Full-Baseline	34.46 (20.90)	30.69 (12.66)
No-Color Preview	6.09 (15.20)	42.64 (18.18)
Color Full-Baseline	8.40 (14.43)	41.11 (19.90)
Color Preview	1.87 (13.51)	38.42 (16.04)
Half-Baseline	-	39.79 (15.05)

An ANOVA on the average slopes as a function of the number of irrelevant elements with the variable Condition (no-color full-baseline, no-color preview, color-full-baseline, and color preview) showed a significant effect of Condition, *F*(2.17, 52.18) = 20.74, *MSE* = 361.98, *p* < .001. Post-hoc comparisons using the Fisher LSD test revealed that the slopes obtained in the no-color preview, the color full-baseline, and the color preview conditions were statistically equivalent to each other (all *p*’s > .158) but less steep than those obtained in the no-color full-baseline condition (all *p*’s < .001).

An ANOVA on the average slopes as a function of the number of relevant elements with the variable Condition (no-color full-baseline, no-color preview, color full-baseline, color preview, and half-baseline) showed a significant effect of Condition, *F*(4, 96) = 2.52, *MSE* = 214.46, *p* = .046. Post-hoc comparisons using the Fisher LSD test revealed that the slopes obtained in the no-color preview, the color full-baseline, the color preview, and the half-baseline conditions were statistically equivalent to each other (all *p*’s > .310). The slope obtained in the no-color full-baseline condition was less steep than that in the no-color preview, the color full-baseline, and the half-baseline condition (all *p*’s < .030), but statistically equivalent to that obtained in the color preview condition (*p* = .065).

#### Error rates

Table 4 depicts the averaged error rates (%) per condition. The average error rate across conditions was 6.0 % and the overall pattern resembled the pattern of RTs. Since there was no indication for any speed-accuracy trade-off, the error rates were not further analyzed.

**Table 4:** Average error rates (%) separately per condition in Experiment 2

	Number of Relevant Elements			
	6		12	
Half-Baseline	4.2		5.6	
	Number of Irrelevant Elements		Number of Irrelevant Elements	
	6	12	6	12
No-Color Full-Baseline	5.5	6.4	8.3	9.0
No-Color Preview	6.3	6.6	9.3	8.1
Color Full-Baseline	4.5	4.3	4.4	6.1
Color Preview	5.1	4.9	5.8	5.7

### Discussion

The results of Experiment 2 show, similar to those of Experiment 1, that a preview reduced the influence of the number of irrelevant elements to the search process in the absence of a color difference but not in the presence of a color difference. Moreover, the plain presence of a color difference was equally effective in reducing the effect of the number of irrelevant elements. However, different from the results of Experiment 1, the search slopes as a function of the number of relevant elements were, apart from that obtained in the no-color full-baseline condition, fully invariant across all conditions, indicating that overall, subset selective search through the relevant elements proceeded in a similar manner to search in the absence of the irrelevant set.

It is interesting to note that the search slope as a function of the number of relevant elements observed in the no-color full-baseline condition tended to be less steep than those in the other conditions. The difference between the no-color full-baseline and the half-baseline condition is particularly remarkable in this respect as the search elements were essentially the same in both conditions. However, search displays in the no-color full-baseline condition contained on average nine more elements than those in the half-baseline condition. Consequently, the overall display size was much higher, which might have facilitated observers to simultaneously reject larger chunks of elements in the no-color full-baseline condition than in the half-baseline condition (Duncan & Humphreys, 1989; Hodsoll & Humphreys, 2007; Poisson & Wilkinson, 1992). Moreover, even though there was again no interaction between numbers of irrelevant and relevant elements, the results depicted in Fig. 3 show that the search slope as a function of the number of irrelevant elements tended to be less steep in the presence of 12 compared to six irrelevant elements in the full-baseline condition. This suggest that observers might have been somewhat more inclined to prematurely terminate the search process in the no-color full-baseline condition than in the other conditions, which in turn might have led to shallower search slopes as a function of the number of relevant elements.

However, irrespective of the reason for the lower search slope in the no-color full-baseline condition, the results of Experiment 2 clearly show that a preview in the presence of a color difference does not contribute to any gain in search efficiency through the relevant elements: search slopes as a function of the number of relevant elements are statistically equivalent across the color full-baseline and the color preview condition. This finding suggests that changes in search efficiency as a function of the number of relevant elements are tightly coupled to the presence of a unique target within the relevant set of elements.

## General discussion

The aim of the present study was to examine the relative contribution of irrelevant and relevant set sizes to preview search in the absence and presence of a color difference between subsets of elements. Two experiments were performed in which numbers of irrelevant and relevant elements were independently manipulated. Furthermore, irrelevant and relevant sets of elements were either simultaneously presented or separated by a preview, and either presented in one color or differently colored, resulting in four conditions: the no-color full-baseline condition, the no-color preview condition, the color full-baseline condition, and the color preview condition. In addition there was a fifth condition, the half-baseline condition, in which only the relevant elements were presented. The target was uniquely distinct within the relevant set of elements in Experiment 1 but not in Experiment 2. There are two major results.

First, the preview benefit as observed in the absence of any color difference between subsets was demonstrated to be entirely due to a reduction in the influence of the irrelevant elements, irrespective of whether the target was defined by a unique feature (Experiment 1) or a conjunction of features (Experiment 2). Second, there was no preview benefit in the presence of a color difference between subsets with a conjunctively defined target (Experiment 2) but there was with a uniquely defined target (Experiment 1). This benefit was shown to be caused by a decline in the influence of the number of relevant elements rather than by a reduction in the effect of the number of irrelevant elements.

The finding that a preview in the absence of any color difference between subsets leads to a reduction in the effect of the number of irrelevant elements is not new. Several studies have found similar results (Al-Aidroos et al., 2012; Donk & Theeuwes, 2001, 2003; Emrich et al., 2008; Jiang et al., 2002b; Kunar, Humphreys, Smith, & Hulleman, 2003; Olivers et al., 1999; Theeuwes et al., 1998) and typically accounted for these results by stating that people can prioritize the selection of new elements by inhibiting the locations of the old elements (Kunar, Humphreys, Smith, & Hulleman, 2003), by temporal grouping (Jiang et al., 2002b), or by being captured by the luminance onsets of the new elements (Donk & Theeuwes, 2001).

However, the results obtained in the presence of a color difference between subsets are more difficult to reconcile with previous studies. The finding that a preview fails to reduce the effect of the number of irrelevant elements is inconsistent with the idea that a preview contributes to a better ability to ignore the irrelevant elements (e.g., Watson & Humphreys, 1997). In fact, search in the color full-baseline condition was already primarily restricted to the relevant elements only. This latter finding is in line with previous findings on color-based selectivity (Egeth et al., 1984; Friedmanhill & Wolfe, 1995; Kaptein et al., 1995; Theeuwes, 1994) and shows that performance in the color full-baseline conditions did not leave much room for further improvement through a reduction in the influence of the number of irrelevant elements. Nevertheless, there are numerous studies reporting profound preview effects with differently colored sets (Braithwaite & Humphreys, 2003; Humphreys, Olivers, et al., 2006; Humphreys et al., 2004; Humphreys et al., 2002; Irwin & Humphreys, 2013; Kiss & Eimer, 2011; Kunar, Humphreys, & Smith, 2003a, 2003b; Kunar, Humphreys, Smith, & Hulleman, 2003; Kunar, Humphreys, Smith, & Watson, 2003; Olivers & Humphreys, 2003; Olivers et al., 1999; von Muhlenen et al., 2013; Watson, 2001; Watson et al., 2008; Watson et al., 2011; Watson & Humphreys, 1997, 1998, 2002; Watson & Inglis, 2007; Watson & Kunar, 2010; Zupan et al., 2015), and, more importantly, these effects were typically attributed to a reduction in the influence of the number of irrelevant elements.

One possible explanation for this discrepancy can be found in the difference in colors used in the present study compared to previous studies on the preview benefit. The large majority of these previous studies used colors that were less different from each other than those used in the present study. For instance, in the original study of Watson and Humphreys (1997), observers searched for a light blue H among a relevant set of light blue As and an irrelevant set of light green Hs. Studies that followed have often adopted the same color combination (Humphreys et al., 2002; Irwin & Humphreys, 2013; Kiss & Eimer, 2011; Kunar, Humphreys, & Smith, 2003a, 2003b; Kunar, Humphreys, Smith, & Hulleman, 2003; Kunar, Humphreys, Smith, & Watson, 2003; Olivers et al., 1999; von Muhlenen et al., 2013; Watson, 2001; Watson et al., 2008; Watson et al., 2011; Watson & Humphreys, 1997, 1998, 2002; Watson & Inglis, 2007; Watson & Kunar, 2010). It is quite possible that similar colors which are close in color space cannot be used to segregate relevant from irrelevant elements as effectively as dissimilar colors, such as those used in the present study (Sobel & Cave, 2002; Williams & Reingold, 2001). Thus, numbers of irrelevant “old” elements might still have had a relative large effect on (color) full-baseline search in these previous studies with the result that this effect could become further reduced by the addition of a preview.

An alternative explanation might be related to the fact that studies reporting preview benefits in the presence of a color difference typically did not independently manipulate the numbers of irrelevant and relevant elements. As a consequence, previously reported preview benefits might actually not have been caused by a reduction in the influence of the number of irrelevant elements but rather by a reduction in the effect of the number of relevant elements. Indeed, studies using differently colored subsets typically used uniquely defined targets, which, as demonstrated by the results of Experiment 1, may lead to a preview benefit which is entirely based on a more efficient search process through the relevant elements. Moreover, several studies even reported a preview benefit with a large color difference between relevant and irrelevant sets of elements (Humphreys, Olivers, et al., 2006; Humphreys et al., 2004; Olivers & Humphreys, 2003; Zupan et al., 2015). As demonstrated here and elsewhere (Egeth et al., 1984; Friedmanhill & Wolfe, 1995; Kaptein et al., 1995; Theeuwes, 1994), the plain presence of a large color difference between subsets already minimizes the influence of irrelevant elements, rendering it unlikely that the observed preview benefit was truly caused by a reduction in the influence of the irrelevant elements. For instance, in a study by Olivers and Humphreys (2003), relevant and irrelevant sets of elements were not only defined by differently oriented lines (i.e., right tilted and left tilted), but also by highly distinct colors (i.e., red and green). In their Experiment 1 observers had the task to search for a red vertical line amongst a relevant set of red right-tilted lines and an irrelevant set of green left-tilted lines, and indicate the position of a black stripe which could be either presented at the top or the bottom of the target line. The relevant set of elements was either presented alone (single-feature condition), simultaneously with the irrelevant set (conjunction condition) or 1 s after the presentation of the irrelevant set (preview condition). The results showed a preview benefit as indicated by the finding that search was more efficient in the preview condition than in the conjunction condition. Moreover, search efficiency in the preview condition was similar to that in the single-feature condition. On the basis of these results, Olivers and Humphreys concluded that the irrelevant elements could be “effectively and efficiently ignored when preceded by a preview display” (see p. 10, Olivers & Humphreys, 2003). However, considering the present results, it is questionable whether this conclusion was justified. Like many studies on the preview effect (Humphreys et al., 2002; Irwin & Humphreys, 2013; Kiss & Eimer, 2011; Kunar, Humphreys, & Smith, 2003a, 2003b; Kunar, Humphreys, Smith, & Hulleman, 2003; Kunar, Humphreys, Smith, & Watson, 2003; Olivers et al., 1999; von Muhlenen et al., 2013; Watson, 2001; Watson et al., 2008; Watson et al., 2011; Watson & Humphreys, 1997, 1998, 2002; Watson & Inglis, 2007; Watson & Kunar, 2010), Olivers and Humphreys did not independently manipulate the numbers of relevant and irrelevant elements but varied the numbers of relevant and irrelevant elements concurrently. It might therefore well have been the case that the observed preview effect was not due to a reduction in the influence of the number of irrelevant elements, but rather to a reduction in the influence of the number of relevant elements.

Indeed, the results in the present Experiment 1 clearly show that if a target is defined by a unique feature relative to the relevant set of elements, a preview benefit in the presence of a color difference is entirely based on a more efficient search process through the relevant elements. Search slopes as a function of the number of irrelevant elements were statistically equivalent across the color preview and the color full-baseline condition, whereas search slopes as a function of the number of relevant elements were substantially reduced in the former compared to the latter condition. Moreover, these relevant search slopes were equivalent across the color preview and the half-baseline condition but substantially steeper in the color full-baseline condition. These findings suggest that the addition of a temporal separation between differently colored subsets may, similar to the addition of a spatial separation (Theeuwes, 1996), actually change search through the relevant elements from serial when subsets only differ in color, to parallel in the presence of an additional preview. Notably, the efficiency gain observed in Experiment 1 could not be replicated in Experiment 2, suggesting that the occurrence of a preview benefit in the presence of a color difference is closely coupled to the specific target-distractor relationship within the relevant set of elements.

An important question that arises is why a preview leads to parallel search only when relevant and irrelevant elements bear distinct colors. That is, why did search through the relevant set of elements proceed in parallel in the color-preview condition but not in the preview condition of Experiment 1? One possibility is that the irrelevant elements were inhibited by visual marking (Watson & Humphreys, 1997) in both conditions, but that the suppression of the irrelevant elements was more effective in the former than in the latter condition by the additional application of color-based inhibition. Accordingly, the presence of a color difference might have augmented the total amount of inhibition applied to the irrelevant elements with the result that the irrelevant elements eventually became virtually absent, which in turn allowed search through the relevant set to change from a serial to a parallel mode. Even though this is theoretically possible, it is questionable whether it is correct to attribute the preview gains observed in the presence of a color difference to more inhibition of the irrelevant elements, for the observed changes are undoubtedly related to a modulation of the search process through the relevant elements only. Alternatively, prioritized selection might have relied on onset capture (Donk & Theeuwes, 2001) in the preview condition, but on both onset capture and color-based inhibition in the color-preview condition. The abrupt luminance onsets accompanying the presentation of the relevant elements might have potentially allowed parallel search among the relevant elements in both conditions, but onset capture typically operates during a limited time-period only. Accordingly, without any color difference, relevant and irrelevant elements might have become increasingly more equivalent to each other as time passed, with the result that in particular in those trials in which the target was not immediately detected, search became serial. When relevant and irrelevant elements differed in color, observers might have additionally applied color-based top-down inhibition. This might not only have prevented the irrelevant elements from being selected but might have also allowed visual search to be conducted in parallel across the relevant set during a longer time interval after the presentation of the relevant set. Even though it is not possible to distinguish between these possibilities on the basis of the present experiments, the observed qualitative change in search mode underpin the idea that there is no such thing as *the preview benefit*. Observed preview efficiency gains vary dependent on the presence of a color difference and the specific target-distractor relationship within the relevant set. Moreover, these variations can reflect changes in the influence of either irrelevant or relevant elements. This suggests that preview search can be based on different mechanisms dependent on the presence of a color difference and the specific target-distractor relationship involved.

Together the present results show that changes in the efficiency of search with a preview may not only be due to a decreased contribution of the number of irrelevant elements but if the target is defined by a unique feature relative to the relevant elements, also to a reduction of the influence of the number of relevant elements. This latter finding points to the possibility that a preview may qualitatively change the search process through the relevant set of elements and emphasizes the importance of the independent manipulation of the number of irrelevant and relevant elements.
